# Emerging Roles of Single-Cell Multi-Omics in Studying Developmental Temporal Patterning

**DOI:** 10.3390/ijms21207491

**Published:** 2020-10-11

**Authors:** Andrea Lopes, Elia Magrinelli, Ludovic Telley

**Affiliations:** Department of Basic Neuroscience, University of Lausanne, 1005 Lausanne, Switzerland; andreavanessa.serralvalopes@unil.ch (A.L.); elia.magrinelli@unil.ch (E.M.)

**Keywords:** central nervous system, development, temporal patterning, intrinsic, extrinsic, neural progenitors, neuron, omics, bioinformatics

## Abstract

The complexity of brain structure and function is rooted in the precise spatial and temporal regulation of selective developmental events. During neurogenesis, both vertebrates and invertebrates generate a wide variety of specialized cell types through the expansion and specification of a restricted set of neuronal progenitors. Temporal patterning of neural progenitors rests on fine regulation between cell-intrinsic and cell-extrinsic mechanisms. The rapid emergence of high-throughput single-cell technologies combined with elaborate computational analysis has started to provide us with unprecedented biological insights related to temporal patterning in the developing central nervous system (CNS). Here, we present an overview of recent advances in *Drosophila* and vertebrates, focusing both on cell-intrinsic mechanisms and environmental influences. We then describe the various multi-omics approaches that have strongly contributed to our current understanding and discuss perspectives on the various -omics approaches that hold great potential for the future of temporal patterning research.

## 1. Background

During CNS development, undifferentiated cells initially divide to generate highly diverse cell types, within and across species. The intricate process implicated in the generation of cellular, structural and functional diversity is regulated in both space and time. Relying on the presence of molecular cues at the interface of brain structures as well as on the presence of gradients, cells assemble to generate complex 3D spatial domains [1,2,3,4]. Temporal progression provides an additional dimension to developmental regulation. Indeed, the production and proper assembly of CNS cells into functional networks rely on the interplay between intrinsic temporally regulated programs and extracellular timing signals. The tight control of these two mechanisms permits the generation of a wide variety of cell types from a restricted and relatively homogeneous set of neural progenitors. Neural progenitor cells undergo temporal fate specification and sequentially produce distinct types of neurons, which progressively populate the developing CNS. One of these temporal transitions occurs upon the proliferative (symmetric) to neurogenic (asymmetric) switch in the division mode of first neuroepithelial cells (NEs) and then apical radial glial cells (aRGs) [5]. While cell-intrinsic temporally regulated programs were long shown to play a main role in the temporal tuning of these events, a growing body of evidence suggests the additional involvement of extrinsic cues.

**Figure 1:**
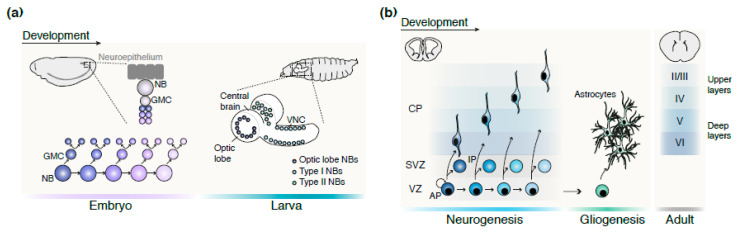
Neural development in Drosophila and in the mammalian cerebral cortex. (**a**) During embryogenesis, NBs delaminate from the neuroepithelium and then divide asymetrically (type I division mode) to produce a GMC. The latter divides once more to produce a pair of neurons that will be successively organized in a laminar manner: the early-born neurons will be located in deeper layers (light purple layer), whereas late-born neurons will be positioned closer to the surface (dark purple layer). At the larval stage, the larval brain is divided into three main regions: the central brain, the optic lobe, and the VNC, each containing various types of NBs. (**b**) During cortical development, excitatory neurons are sequentially generated directly from APs or indirectly from IPs. Neurons born earlier during development will settle in the deep layers of the adult cortex, whereas the upper layers will be composed of later-born neurons. At the end of the neurogenic period, APs undergo a self-consuming division to generate glial cells. Abbreviations: AP, apical progenitors; CP, cortical plate; GMC, ganglion mother cell; IP, intermediate progenitors; NB, neuroblast; VNC, ventral nerve cord; VZ, ventricular zone; SVZ, subventricular zone.

In this review, we will discuss key principles defining the temporal progression of cell identity and the extracellular timing signals driving CNS development. We will first summarize the general mechanisms underlying temporal progression in identity through a detailed analysis of *Drosophila* CNS and mouse corticogenesis development (Figure 1). We will then describe multi-omics and bioinformatic models that best address this process and highlight the major pending questions.

## 2. Overview of CNS Neurogenesis

### 2.1. Drosophila Ventral Nerve Cord Neurogenesis

The *Drosophila* CNS comprises the central brain, the ventral nerve cord (VNC) and the optic lobe (OL) (Figure 1a). The VNC is arguably best described and will be used as a reference structure here. The embryonic VNC is segmented into a series of repeated units termed neuromeres; each is divided into two hemisegments. Each hemisegment of the VNC is composed of approximately thirty unique neural stem cells (NSCs) or neuroblasts (NBs, an asymmetrically dividing NSC) organized in rows and columns [6]. Each NB, independent of its location in the nervous system, gives birth to a stereotypical series of neurons via three different types of asymmetric divisions. Thus, the NB population is self-renewed upon each division, additionally yielding a distinct cell type: (i) Type-0 divisions directly give birth to a differentiating neuron, (ii) Type-1 divisions produce ganglion mother cells that further divide once into two neurons each, and (iii) Type-2 divisions generate intermediate neural progenitors (INP), which, in turn, further give rise to ganglion mother cells over a prolonged series of asymmetric divisions [7,8,9]. Neurogenesis occurs in two sequential waves: (i) during the embryonic phase and (ii) throughout the larval and pupal stages, in preparation for the final metamorphosis [10,11]. Embryonic neurogenesis is a one-day long event during which distinct NB types transition through a cascade of temporal transcription factors (tTFs) giving rise to a series of restricted lineages. The short length of this neurogenic wave underlies the precise NB-intrinsic temporal progression. At the end of embryogenesis, the majority of NBs undergo apoptosis, while few enter G1 quiescence [12]. By the beginning of the larval stage, surviving NBs exit quiescence and initiate a second, longer wave of neurogenesis, spanning both the larval and pupal stages [10,11,13].

### 2.2. Mouse Neocortex Neurogenesis

The developing mouse neocortex emerges within the dorsal pallium (Figure 1b). Here, NEs undergo symmetric divisions to amplify the pool of aRGs in the juxtaventricular germinal zone called the ventricular zone (VZ). Next, aRGs initiate neurogenesis through a series of asymmetric divisions. The first neurogenic events are dominated by direct neurogenesis, during which aRGs directly produce excitatory neurons. This phase is followed by the emergence of indirect neurogenesis, generating intermediate transiently amplifying progenitors (IP) [14,15]. These cells are located dorsally to the VZ in the subventricular zone (SVZ). Gliogenesis follows corticogenesis when aRGs undergo self-consuming symmetric divisions to generate glial cells [16]. Meanwhile, ventral pallium-derived interneurons migrate tangentially and ultimately populate the neocortex. Readers further interested in this topic can refer to a recent detailed review [17].

Our current understanding of the mechanisms of mouse neocortical temporal progression is still significantly restricted in comparison to the *Drosophila*. However, several studies have thoroughly described the stereotypical aRG transition from deep to superficial layer neuron-producing cells [16,18]. Recent studies have identified a core set of genes and postmitotic transcriptional events crucial to determine the final fate of differentiating neurons [19]. On the other hand, the timing of gliogenesis has not been clearly associated with a precise laminar position preference, as the majority of astrocytes are born from grey matter localized proliferation, with a small degree of 3D dispersion from their clonal origin [20,21].

## 3. Regulation of CNS Temporal Patterning

Much of our current knowledge on the temporal controls over the generation of neuronal diversity is derived from studies in the fruit fly *Drosophila melanogaster*. Indeed, temporal patterning was first described in *Drosophila* VNC NBs [22,23,24], occurring at both the embryonic and larval stages, during which several important temporal transitions take place. The successive expression of tTFs during precise temporal windows is necessary and sufficient to specify distinct neuronal fates. Unlike vertebrates, all these mechanisms have long been demonstrated to rely on progenitor-intrinsic programs via transcriptional cross-regulation between tTFs, along with other independent mechanisms [22,23,24,25,26]. However, recent studies have revealed the additional role of extrinsic cues in the regulation of *Drosophila* NB patterning, thus pointing in the direction of evolutionarily conserved strategies shared by both invertebrates and vertebrates.

### 3.1. Epigenetic and Transcriptional Events Regulating Phases of Temporal Progression

Understanding how cells measure and react to the progression of time (temporal progression) is not straightforward. Considering the *Drosophila* nervous system development, a limited set of feed-forward cross-regulated transcription factors (TFs) appears to be sufficient to generate a reproducible temporal sequence of transcriptional events [27,28,29,30,31]. For the sake of brevity, we will exclusively highlight a few examples of temporal patterning progression in the *Drosophila* CNS to extrapolate the general mechanisms of cell-autonomous temporal patterning. For further descriptions, we refer the readers to these reviews [13,27,32].

As already described for a series of biological events [33,34,35], the synchronization of patterning events requires the existence of molecular timers in each cell [36]. During *Drosophila* CNS development, this process has often been described as a series of activating timers. Here, temporal progression is paced by the expression and accumulation of the tTF itself. When a needed threshold is reached, the expression of the next activator in the cascade is induced. Thus, Pdm expression onset is mostly determined by the transcription of Kr, which is itself activated by Hb. Pdm expression would, in turn, determine the activation of the Cas-expressing phase. Isolated early- and late-forming NB lines are capable of recapitulating the sequential gene expression of Hb → Kr → Pdm → Cas, supporting the hypothesis of a coordinated action between gene regulatory interactions with an independent cell-intrinsic molecular temporal clock [24].

**Figure 2:**
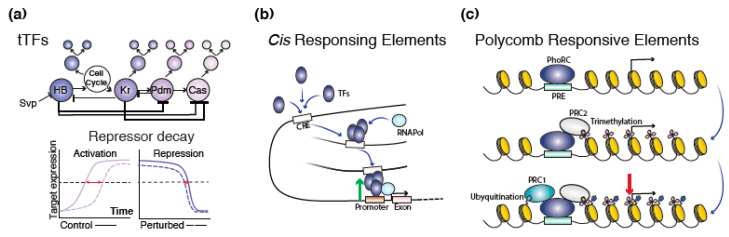
Epigenetic and transcriptional regulation of temporal progression. (**a**) During Drosophila CNS development, temporal progression can be paced by two possible scenarios: the accumulation of a tTF or by the decay of upstream tTF repressors. (**b**) In the developing Drosophila, gene transcriptional activation (green arrow) can be achieved through the binding of TFs onto CREs. (**c**) PREs mediate gene silencing (red arrow) by recruiting polycomb complexes. Abbreviations: CRE, cis regulatory elements; TFs, transcription factors; tTFs, temporal transcription factors; PRE, polycomb responsive elements; PhoRC, pleiotropic repressive complex; PRC1, polycomb repressive complex 1; PRC2, polycomb repressive complex 2.

Recent evidence suggests that a more robust regulatory mechanism of the stereotypical temporal patterning cascade in VNC NBs occurs by means of the decay of tTF repressor timers [37] (Figure 2a). In this case, temporal progression is more sensitive to the upstream expression of a tTF repressor rather than the expression of activating timers. As the upstream repressor expression decays to a sufficiently low level, NBs transition in the following temporal step, representing a permanent and less costly mechanism for gating temporal progression [38]. As an example, Pdm expression regulation is more sensitive to the upstream repressor Hb than to the deletion of the upstream activator Kr. Similarly, Cas expression timing is advanced in Kr mutants, in contrast to Pdm mutants [37].

The tight control of gene transcription depends upon the binding of TFs onto *cis* regulatory elements (CREs) (Figure 2b), from which transcription initiation complexes are recruited. As shown in the developing *Drosophila*, many tTF-CRE pairs are required for temporal regulation [28,29]. For instance, three evolutionarily conserved regulatory regions within the Hb enhancer region (HG4-1, HG4-3 and HG4-7) have been functionally confirmed to specifically recapitulate Hb expression in NB stages 9 and 10, but not in stage 11 [28].

On the other hand, gene silencing in *Drosophila* is mediated through the recruitment of the Polycomb complex by DNA elements termed Polycomb responsive elements (PRE) [39] (Figure 2c). PREs have been implicated in the repression of hundreds of developmentally relevant *loci,* many of which are widely conserved across vertebrates and invertebrates, and include several developmental key regulators, such as *Hox* genes [40,41]. Interestingly, a recent study demonstrated the localization of the *Drosophila* pleiotropic repressive complex (PhoRC) in both enhancers and PRE elements during development. Here, BiTS-ChIP-seq (batch isolation of tissue-specific chromatin for immunoprecipitation) and high-throughput sequencing in *Drosophila* embryonic tissue was used to investigate the association of PRE elements with genes in the active state of transcription and further confirmed when isolated in a reporter construct [42]. In light of these results, the Polycomb complex and TFs appear to be in direct competition for gene regulation.

Temporal progression can also be controlled by limiting the number of physically available DNA regions. Thus, epigenetic modifications control TF activity by regulating accessible and inaccessible chromatin regions (reviewed in [43]). The modifications occurring in the chromatin landscape during neural differentiation transitions have been studied in both *Drosophila* [44] and murine stem cells [45].

A complete and highly detailed map of the 3D chromatin structural changes characterizing neuronal differentiation in mouse neural cell lines has also been generated [45]. These findings highlight chromatin remodelling during neuronal differentiation, including at the small-scale level of topologically associated domains (TADs). Here, chromatin accessibility is favoured across active regions by decreasing the strength of TAD interactions [45]. 

In vertebrates, chromatin accessibility is further regulated by two distinct chromatin-modifying complexes: Polycomb repressive complex 1 (PRC1) and Polycomb repressive complex 2 (PRC2). PRC2-mediated gene-silencing occurs through its enzymatic subunits EZH1 and EZH2, which methylate (di- and tri-) the Lys residue of histone H3 (H3K27me2/3). The PRC1 complex catalyses the monoubiquitylation of Lys 119 of histone H2A (H2AK119ub) by the RING finger domain and was implicated in gene regulation by compacting chromatin in an enzymatic activity-independent manner [46,47]. Neurogenic competence was shown to be regulated by both complexes as the removal of RING1B and EZH2, in the developing cortex, prolonged the neurogenic phase, hence delaying the onset of astrogenesis [48]. PRC2 repressive function is also implicated in the fine-tuning between self-renewal and differentiation of cortical progenitor cells. By removing EZH2 expression prior to the onset of neurogenesis, cortical development was characterized by an acceleration of the developmental timing, accompanied by a drastic reduction in the neuronal output and up-regulation in gene expression [49]. A recent study provided additional evidence on its involvement in the epigenetic regulation of temporal progression. In this case, H3k27 histone modification by PRC2 was shown to control mouse aRG temporal progression [19]. Single-cell RNA sequencing (scRNA-seq) was performed on isochronic cohorts of mouse aRGs at various developmental time points, identifying a specific early expression of PRC2 components during corticogenesis. To functionally address the role of this complex, a knockout mouse was generated to abrogate the expression of PRC2 specifically in the cortex. Loss of function resulted in an anticipation of the neurogenic program, therefore reducing the total corticogenic period and the overall cortical thickness [19].

### 3.2. Cell Cycle as a Switching Event in Temporal Patterning

During CNS development, a key readout for the temporal progression of progenitors is the identity of daughter cells. Yet, the contribution of the cell cycle to this process remains elusive. Several observations have excluded the cell cycle as the main mode of determining tTF progression. Even during stereotypical and fast-paced *Drosophila* VNC development, each tTF is expressed for a variable number of cell divisions, from a single division for *Hb* to two or three divisions for other factors [13,24,50]. In addition, NBs can still recapitulate the typical tTF expression sequence when isolated in vitro and arrested in G2, after reaching the *Kr* expression stage. In light of these results, cell cycle progression appears to be exclusively required by the temporal steps preceding *Kr* expression [25,51,52,53].

Although the progression of temporal identity does not primarily rely on the cell cycle, it is not completely independent of it. Indeed, temporal progression in NBs serves the purpose of regulating the cellular output resulting from cell division. tTF expression may nonetheless reflect cell cycle events. Such a mechanism has already been described with the activation of one tTF regulator, the orphan nuclear receptor *Svp (Nr2f1/2* paralog). *Svp* is transcribed during cytokinesis and represses *Hb*, which, in turn, promotes temporal progression in cellular identity acquisition [26,54].

The overall uncoupling of the cell cycle and temporal progression is also observed in mammalian cortical aRGs, the main self-renewing progenitors of the developing cortex [55]. These progenitor cells progress through a temporal program, even upon cell cycle arrest in vivo [55]. Indeed, the temporal transcriptional signatures were maintained in aRGs even when cell cycle arrest was forced via electroporation of the Cdk2 inhibitor [55].

### 3.3. Extracellular Regulation of Temporal Patterning

**Figure 3:**
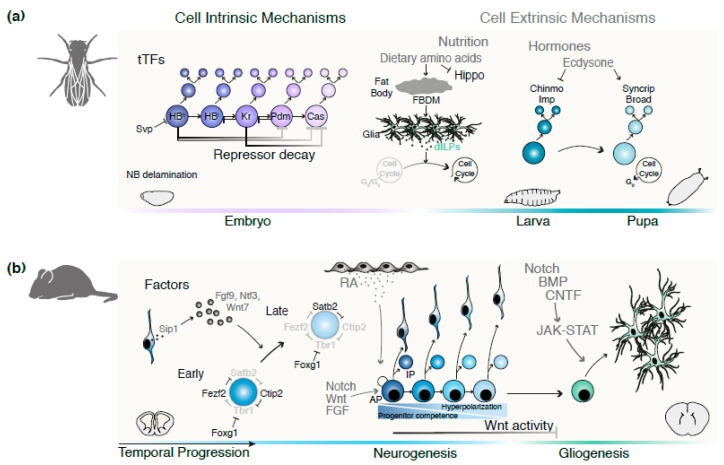
Temporal patterning in Drosophila and in the mammalian cerebral cortex. (**a**) Illustration of the cell-intrinsic and -extrinsic temporal patterning events that take place in the developing Drosophila. During embryogenesis, neuroblasts transition through a cell-autonomous, temporally regulated tTFs cascade to generate distinct neuronal subtypes. In the early larvae, extrinsic cues exert their influence by mediating the exit from quiescence and consequent cell cycle re-entry via nutritional-dependent stimulus. The later temporal switch from early to late tTFs is triggered by extrinsic hormonal cues, in the late larvae stage. (**b**) Summary of the cell-intrinsic and -extrinsic controls on the temporal progression of mammalian progenitors. During corticogenesis, a six-layer neocortex is generated by the direct or indirect production of neurons from APs or IPs, respectively. The restricted core of TFs drives the deep versus superficial layer identity via cross-regulation. As development proceeds, the transition in neuronal identities is accompanied by the progressive decrease in progenitor competence, hyperpolarization of APs and a switch from a neurogenic to a gliogenic state under the influence of signaling pathways. Abbreviations: AP, apical progenitors; dILPS, Drosophila insulin-like peptides; FBDM, fat body-derived mitogen; IP, intermediate progenitors; NB, neuroblast; RA, retinoic acid; tTF, temporal transcription factor.

In contrast to embryonic stages, the regulation of postembryonic neurogenesis in *Drosophila* involves progenitor-extrinsic signals [56] (Figure 3a). Indeed, recent lines of evidence have shown extrinsic controls over the developmental events at play during *Drosophila* larval stages. The properly timed exit from quiescence and cell cycle re-entry of larval NBs are the first events that are partly regulated by extrinsic cues via nutrition-dependent stimuli.

During larval feeding, the liver-like fat body starts secreting a growth factor/mitogen (FBDM) upon the detection of dietary amino acids [57]. In response to these signals, neuroblast-neighbouring subperineurial glial cells (SPGs) produce insulin/insulin-like growth factor (IGF) peptides (dILPs), thus driving NB reactivation [58,59].

Suppression of the cell growth inhibitor Hippo pathway is an additional mechanism through which nutritional cues and glia prompt NB enlargement and cell cycle re-entry. Both NBs and glial cells express the cell surface transmembrane proteins Crumbs and Echinoid that are necessary to activate the Hippo pathway. As a result, NB enlargement and proliferation are hampered. The loss of Crumbs, Echinoid or the core kinase *hippo* is sufficient to trigger premature NB reactivation from quiescence, therefore stimulating proliferation [60,61]. Glia also negatively regulate the proliferation of NBs by secreting the *Drosophila* Anachronism (Ana) glycoprotein. The mechanism through which NB quiescence is maintained remains elusive [62].

In addition to nutrition and glia-dependent regulation, temporal patterning of embryonic and larval NBs by extrinsic hormonal cues has been demonstrated in recent studies. The steroid hormone ecdysone is the key regulator that mediates an early-to-late gene expression transition in central brain type II NBs. During early larval stages, Chinmo- and Imp-expressing INPs give rise to neurons and glia. In contrast, later larval lineages solely produce neurons and express Broad and Syncrip. By ensuring the down- and upregulation of the early and later expressed genes, respectively, the ecdysone signalling pathway contributes to the neuronal diversity produced by each larval NB [26].

As previously discussed, in the developing mammalian neocortex, cell-intrinsic temporal programs are crucial for the formation of functional radial units. However, growing evidence further suggests the implication of extrinsic cues on the temporal “tuning” of these events (Figure 3b). For example, fibroblast growth factor 2 (FGF2) promotes cortical progenitor proliferation, leading to increased neuronal and glial output and expansion of the cerebral cortex [63]. Similarly, the Wnt/β-catenin signalling pathway stimulates proliferative divisions, expanding the precursor populations [64]. In line with these results, a recent study demonstrated that inhibition of the Wnt/β-catenin pathway driven by progenitor hyperpolarization promotes a forward shift in the temporal progression of aRG division modes [65]. Meninge-derived retinoic acid (RA) has been suggested to participate in the regulation of progenitor division. The loss of RA signalling in Foxc1 mutants, which lack forebrain meninges, results in prolonged proliferative divisions. Thus, cortical development is hampered by the reduced provision of intermediate progenitors (IPs) and neurons [66]. Finally, the Notch signalling pathway perturbs the neurogenic period by inhibiting neurogenesis [67]. The lack of some effectors involved in Notch signalling leads to premature production of neurons at the expense of neural precursors [68].

By the end of the neurogenic period, progenitors undergo a final temporal transition and switch from neurogenesis to gliogenesis [5]. Several environmental cues act in concert to regulate the appropriate timing of gliogenesis onset [69]. In vitro experiments in which cortical precursors were cultured on embryonic or postnatal cortical slices intriguingly led to the production of neurons and astrocytes, respectively. In this regard, environmental temporal cues appear to strongly influence the neurogenic-to-gliogenic transition [70]. Moreover, several members of the IL-6 neurotrophic cytokine subfamily play a central role in gliogenesis initiation. Through interaction with several coreceptors, the JAK-STAT pathway is consequentially activated, ultimately triggering gliogenesis [71,72,73,74]. The functional interaction between bone morphogenic proteins (BMPs) and Notch ligands together with the aforementioned JAK-STAT pathway also favours the gliogenic switch. The activation of downstream TFs by BMPs promotes the transcription of glial genes, such as *Gfap*, thus inducing gliogenesis [75,76,77]. Notch signalling is a well-documented pathway involved in the temporal transition from neurogenesis to gliogenesis. The neurogenic-to-gliogenic switch may occur either via activation of the *Gfap* promoter or by repression of neurogenic bHLHs by Hes proteins when the JAK-STAT pathway is conjointly activated [78,79]. Altogether, these studies unambiguously demonstrate a relevant contribution of cell-extrinsic signals in the temporal course of corticogenesis.

### 3.4. Perspective on Single-Cell Omics Approaches to Investigate CNS Temporal Patterning

Studies in *Drosophila* have provided most of our understanding of temporal progression and have inspired parallel investigations on vertebrate models. In recent years, the advent of single-cell omics approaches has provided access to the complexity of vertebrate systems thanks to high-throughput sampling. In this last chapter, we will discuss these methods and their potential for better understanding vertebrate temporal progression, most notably in the neocortex.

#### 3.4.1. Single-Cell RNA Sequencing

The wish to decipher the relation between genotypes and phenotypes has long been present in biology and medicine. In light of this interest, next-generation sequencing technologies underwent important progress in the recent years. As our understanding of cellular complexity and variability advanced, conventional bulk sequencing was subsequently adapted towards transcriptome sequencing at the single-cell level. Almost ten years ago, scRNA-seq started to be used to interrogate the transcriptomic profile associated with single isolated cells. For example, this approach was recently applied to identify and functionally characterize neuron-specific primordial transcriptional programs, as they dynamically unfold during neuronal differentiation [80]. In this study, researchers isolated neurons born at specific time points in the developing neocortex using the FlashTag technique [81], followed by scRNA-seq. A similar strategy was posteriorly used to investigate the temporal progression of cortical development by identifying evolutionarily conserved, temporally patterned genes driving aRGs from internally directed to exteroceptive states [19]. In accordance with other studies, the translation of laminar-specific gene mRNA in progenitors was shown to be halted until their further progression in differentiation. Consequently, progenitor daughter cells are transcriptionally primed into a specific differentiation path [82].

A similar approach has also been used in developing *Drosophila* to characterize the transcriptional profiles assigned to specific NB types [83]. NB-specific permanent labelling and scRNA sequencing of the ALad1, ALl1, ALv1 and ALv2 Antennal lobe NB lines and the R14D11 and R41D10 Mushroom body NB lines identified a common neuronal fate-enriched set of 75 TFs as well as 68 mushroom body- and antennal lobe-specific TFs, whose expression patterns were previously unknown. More recently, a comparable method was applied to track time-specific diversity across NB lines [84]. Intermediate and ventral column-derived cells and NBs, neurons and glia were isolated at four independent, 2-hour split time intervals using the DIV-MARIS protocol. Here, in addition to confirming the spatiotemporal pattern of already known protein-encoding temporally specific genes, the authors identified strong spatiotemporal patterning in long noncoding RNA (lncRNA). These data highlight the important role these molecules could play during critical early neurogenic stages.

#### 3.4.2. Spatial Transcriptomics

Temporal patterning often results from its interaction with spatially regulated factors across the tissue. However, most scRNA-seq techniques lead to the loss of spatial information. The need for combining transcriptomic and spatial information led to the emergence of spatial transcriptomics, in which a high number of mRNA molecules can be measured with various degrees of spatial resolution. For example, in Slide-seq [85], RNA molecules are transferred from the tissue on a slide covered in uniquely barcoded microbeads of 10 µm diameter, defining its spatial resolution limit. The microbeads are then covered with DNA oligonucleotides that can bind to 3′ RNA poly-A ends [85], allowing cDNA amplification and sequencing. Requiring up to 10-μm-thick cryosections, this technique provides a clear enough resolution to distinguish monocellular layers, such as ependymal cells from the hippocampus [85]. Additional methods based on the multiplexed variation of single-molecule FISH, such as MERFISH and seqFISH [86,87,88,89], provide high sequencing depth and subcellular resolution. In both of these methods, a set of probes covering the full transcriptome is designed and subsequently sequenced in multiple sequential rounds of hybridization.

#### 3.4.3. Single-Cell ATAC Sequencing

As previously discussed, both *cis* elements and epigenetic states have important intrinsic roles in the complex temporal patterning. A precise mapping of *cis* regulatory elements along with temporally controlled expression or repression of tTFs during cortical development has not yet been performed.

High-throughput chromatin investigation has been implemented in the context of in vitro models for mammalian stem cells undergoing neuronal differentiation [45]. In this case, mouse embryonic stem cells, neuronal progenitors and cortical neurons were used to perform RNA-seq, ATAC-seq and Hi-C. The progressive maturation towards a neurogenic and neuronal program was investigated by characterizing associated regulatory elements and structural signatures [45]. Both examples used bulk methods to analyse the chromatin accessibility and structure associated with transcriptional profiles, finding interesting chromatin structural signatures for specific developmental trajectories. The main drawback of this approach is the requirement of a homogeneous cellular population from which the molecular material is acquired. Despite its success, it reduces the possibly high and significant heterogeneity of developing cell populations, often subjected to dynamic changes. Thus, translating ATAC-seq towards a single-cell specificity could resolve such complexity [90,91].

Therefore, the implementation of multimodal modelling by combining scRNA-seq and scATAC-seq could provide a more comprehensive picture of single-cell diversity [92]. The systematic investigation of chromosomic structure modification during cortical development will eventually reveal the regulatory elements controlling the temporal progression of such complex systems.

#### 3.4.4. Lineage Barcoding

Studies on the temporal patterning of the *Drosophila* CNS have provided a detailed description of the robust association of lineages and the respective neuronal types generated. On the contrary, this lineage relationship has not yet been fully addressed in vertebrate neuronal development. Indeed, clear evidence of the generation of diverse cortical cell types through specific lineages is lacking. Future endeavours should focus on identifying single-lineage derived cells, in the context of single-cell transcriptomic studies, by assigning inheritable barcodes to originally labelled aRGs.

New technologies now allow such complex cellular lineage reconstructions [93]. Readers can refer to a recent detailed review on this topic [94]. One recent example is the genetically modified lineage tracing MARC1 mouse line [95]. Based on CRISPR/CAS9 principles, this mouse line incorporates in its genome a set of loci that are translated into self-homing guide RNA molecules constitutively expressed. In the presence of the CAS9 protein, the self-homing guide RNA will drive CAS9 to target their own loci, inducing random mutations that will be inherited by all further generated daughter cells. Random mutations will likely disrupt the self-homing guide RNA properties, preventing further mutations of each locus. Due to the multitude of randomly mutating loci and the diversity in their mutation efficiency, some mutate very fast, while others do so only after multiple days. As a result, each cell in the lineage will yield a unique mutation profile able to recapitulate its lineage history. Further improvement in the experimental design of such approaches remains an important challenge, as the full reconstruction of the cell type lineage history will only be achieved once these mutation profiles are compared at the single-cell level.

#### 3.4.5. Toward High-Throughput Single-Cell Proteomics and Metabolomics

A major advantage of proteomic analysis is the more accurate reflection of the biological processes ongoing in the cell. Most proteomic techniques have so far been performed in bulk analysis, shedding a light on the association of proteins and post-translational modifications with specific functions and pathologies [96,97]. However, major efforts employed in improving the sensibility and multiplexing of these techniques are revolutionizing the nascent field of single-cell proteomics.

One branch of proteomic analysis focuses on the identification of proteins thanks to antibodies. Miniaturized enzyme-linked immunosorbent assays (ELISA) can quantitatively identify protein levels at the single-cell resolution, targeting up to 40 proteins over tens of thousands of cells [98,99,100]. Alternatively, cytometry-based methods (e.g., Fluorescence flow cytometry, mass cytometry) can semi-quantitatively detect around 30 proteins within a single-cell suspension [101,102]. Interestingly, by combining a similar approach with scRNAseq, researchers developed a method for analysing high-throughput single-cell transcriptome and protein abundance for a few selected targets [103].

Mass spectrometry-based methods (e.g., Time of Flight, Electro-Spray Ionization, Orbitrap) give more high-throughput proteomic analysis, identifying up to thousands proteins for each sample by their respective weight [104]. A reduction in working volumes and further multiplexing would give access to single-cell resolution, thus representing a challenge currently being tackled by multiple independent laboratories [105,106,107,108,109].

Recently, researchers have directly applied mass cytometry to histological preparations, providing spatial information to proteomics analysis. Thus, histological preparations are labelled with heavy metal tagged antibodies and measured by mass cytometry upon laser ablation, allowing single-cell and subcellular resolutions. On top of that, this method has also been successfully coupled to RNA single-molecule imaging. In this case, the analysis of both RNA and protein in the same sample was shown to be possible by simultaneously combining single-cell detection of proteins and mRNA for a few target genes and proteins [110,111].

Beyond proteomics analysis, mass spectrometry serves as a appropriate strategy for analyzing metabolites abundance, such as amino acids, sugars, and lipids [112]. These molecules give direct information about ongoing biological processes [113]. Metabolite structures are highly heterogeneous, have a fast-turnover and are currently largely uncharacterized, making their analysis and sample preparation particularly arduous. Therefore, several methods have been recently developed to generate single-cell high-throughput and spatial metabolomics datasets [113,114,115]. In the not-so-distant future, technological advances could extend our ability to describe the heterogeneity of cell types beyond genes and proteins, allowing researchers to tailor their analysis for the modality according to their questions and needs.

#### 3.4.6. Integrating Datasets

**Figure 4:**
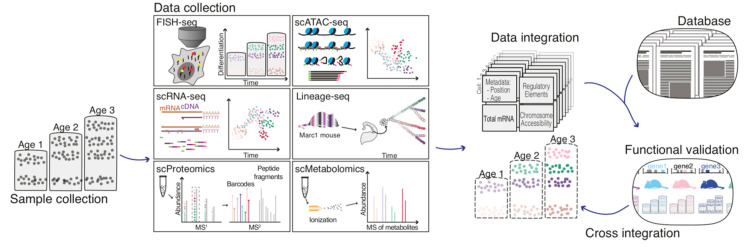
Multi-omics data integration for in silico reconstruction of temporal progression in cortical development. Samples are gathered at multiple time points during cortical development. Independent experiments are performed accessing information from spatial transcriptomics (e.g., FISH-seq), deep single-cell transcriptomic (e.g., scRNA-seq), single cell chromatin accessibility (e.g., scATAC-seq), lineage-seq (e.g., Marc1 mouse), proteomics and metabolomics and ultimately cross referenced with literature data and functional validation. Finally, the cell identities observed can be aligned across different methods, reconstructing emblematic characteristics of single-cells across temporal progression.

The size of omics studies and databases has been expanding at a rapid pace, progressively increasing the variety of structures, conditions, time points and species analysed. Developing methods able to unify heterogeneous datasets is therefore an important current topic in the -omics field. Few methods manage to combine different datasets without compromising the integrity and interpretation of the data. For instance, it is crucial to correct for batch effects when comparing different studies, while still maintaining the feature diversity within the same dataset [116,117,118,119]. The LIGER method tries to circumvent this issue by employing integrative non-negative matrix factorization (iNMF) to identify shared and dataset-specific factors, i.e., genes defining a particular cell type. The dataset-specific effects are then adjusted by a tuning parameter, which reflects the differences in the analysed datasets. Another interesting method is based on the mutual nearest neighbour (MNN) matching, which corrects datasets by recognizing cells that cluster the closest between distinct datasets [116]. Implemented together with canonical correlation analysis (CCA), employed in Seurat V3.0, it determines groups of genes with similar differential expression across batches. Vectors of difference are then used to determine and batch correct anchor points to further transform the datasets [117]. However, the remaining key challenge consists in developing integrative computational tools able to integrate multiple datasets with different experimental contexts and measurement modalities (Figure 4, Table 1). The emergence of these new tools will offer many exciting opportunities in single-cell biology.

## 4. Conclusions

Temporal regulation over the biological processes that underlie the accurate assembly of a functional CNS is becoming increasingly understood. This temporal regulation is subject to fine-tuning by cell-intrinsic and extrinsic mechanisms. As discussed in this review, pioneering research in the *Drosophila* system has facilitated major advances in the identification of cell-intrinsic dependent means through which NBs specify distinct neuronal types and generate neuronal diversity [23,24,27,29,122].

In contrast to *Drosophila*, the temporal dynamics through which mammalian progenitors progressively induce distinct neuronal fates remain poorly understood. Further investigations on the epigenetic mechanisms through which temporal progression is regulated will also deepen our understanding of cell-intrinsic aspects.

In addition to cell-intrinsic temporal regulation, deciphering the role of extrinsic modulation on temporal patterning is fundamental to understanding CNS assembly. The major challenges faced are the loss of surrounding environmental and spatial information that occurs when investigating these complex mechanisms using in vitro systems. Improved methods, in which omics analyses are combined with functional assays while maintaining cellular location, will provide significant insights into the extrinsic controls of temporal patterning. The rapid emergence of new single-cell omics approaches and the growing number of available scRNA-seq datasets have revealed the progenitor transcriptomic profiles over the course of development [19,120]. In silico analyses consisting of integrating several sources of single-cell omics datasets with additional functional analysis may help overcome the above limitations. The integration of multifaceted datasets represents a critical step in understanding and predicting cellular trajectories in the developing CNS.

## Figures and Tables

**Table 1:** Main advantages and disadvantages of the various omics techniques.

Methods	Advantages	Disadvantages	References
scRNA-seq	Measures gene expression at the single-cell resolution; Straightfoward protocol; Available benchmark analysis; Compatible with scATAC-seq	Static snapshot of cell state; Loss of spatial information	[19,80,82,83,84,120]
Spatial transcriptomics	Spatially resolved gene expression	Static snapshot of cell state; Restricted target number of genes; Tedious protocol and analysis	[85,86,87,88,89]
scATAC-seq	Mapping of the accessible chromatin regions at the single-cell resolution; Straightfoward protocol; Available benchmark analysis; Compatible with scRNA-seq	Static snapshot of the current state; Loss of spatial information; Limited starting material	[90,91]
Lineage reconstruction	Mapping of cellular ontogeny	Detectability; Benchmark analysis not available	[93,95,121]
scProteomics	More accurate reflection of a cell state	Static snapshot of the current state; Unstable starting material; Benchmark analysis not available; Limited access to the technology	[98,101,105,110]
scMetabolomics	Direct readout of ongoing biological processes; Closest to the functional phenotype	Static snapshot of the current state; Lack of standardized data repository; Benchmark analysis not available; Limited access to the technology	[112,113]
Spatial proteomics/metabolomics	Spatially resolved proteomic and metabolomic expression	Limited access to the technology	[111,115]

## Abbreviations

AP	Apical progenitor
aRG	Apical radial glia
BMP	Bone morphogenic protein
Cas	Castor
CCA	Canonical correlation analysis
CNS	Central nervous system
CP	Cortical plate
CRE	Cis regulatory element
dILPs	Insulin-like growth factors like peptides
E	Embryonic day
FGF2	Fibroblast growth factor 2
FISH	Fluorescence in situ hybridization
GMC	Ganglion mother cell
Hb	Hunchback
iNMF	Integrative non-negative matrix factorization
INP	Intermediate neural progenitors
IP	Intermediate progenitors
Kr	Krüppel
LIGER	Linked inference of genomic experimental relationships
LncRNA	Long noncoding RNA
MNN	Mutual nearest neighbor
NB	Neuroblasts
NEs	Neuroephitelial cells
NSC	Neural stem cells
OL	Optic lobe
PRC1	Polycomb repressive complex 1
PRC2	Polycomb repressive complex 2
PRE	Polycomb responsive element
PhoRC	Pleiotropic repressive complex
RA	Retinoic acid
scRNA-seq	Single-cells RNA sequencing
scATAC-seq	Single-cellsSingle cell Assay for Transposase-Accessible Chromatin with high throughput sequencing
SPG	Subperineurial glial cells
SVZ	Subventricular zone
TAD	Topologically associating domains
TF	Transcription factor
tTF	Temporal transcription factors
VNC	Ventral nerve cord
VZ	Ventricular zone.
